# Pediatric cancer—pathology and microenvironment influence: a perspective into osteosarcoma and non-osteogenic mesenchymal malignant neoplasms

**DOI:** 10.1007/s12672-024-01240-5

**Published:** 2024-08-18

**Authors:** Consolato M. Sergi

**Affiliations:** 1 Division of Anatomic Pathology, Department of Laboratory Medicine, Children’s Hospital of Eastern Ontario (CHEO), University of Ottawa, 401 Smyth Road, Ottawa, ON K1H 8L1 Canada; 2 Department of Laboratory Medicine, Stollery Children’s Hospital, University of Alberta, Edmonton, AB Canada; 3 University of Ottawa, Ottawa, ON Canada

**Keywords:** Tumor microenvironment, Epithelial-to-mesenchymal transition, Mesenchymal-to-epithelial transition

## Abstract

Pediatric cancer remains the leading cause of disease-related death among children aged 1–14 years. A few risk factors have been conclusively identified, including exposure to pesticides, high-dose radiation, and specific genetic syndromes, but the etiology underlying most events remains unknown. The tumor microenvironment (TME) includes stromal cells, vasculature, fibroblasts, adipocytes, and different subsets of immunological cells. TME plays a crucial role in carcinogenesis, cancer formation, progression, dissemination, and resistance to therapy. Moreover, autophagy seems to be a vital regulator of the TME and controls tumor immunity. Autophagy is an evolutionarily conserved intracellular process. It enables the degradation and recycling of long-lived large molecules or damaged organelles using the lysosomal-mediated pathway. The multifaceted role of autophagy in the complicated neoplastic TME may depend on a specific context. Autophagy may function as a tumor-suppressive mechanism during early tumorigenesis by eliminating unhealthy intracellular components and proteins, regulating antigen presentation to and by immune cells, and supporting anti-cancer immune response. On the other hand, dysregulation of autophagy may contribute to tumor progression by promoting genome damage and instability. This perspective provides an assortment of regulatory substances that influence the features of the TME and the metastasis process. Mesenchymal cells in bone and soft-tissue sarcomas and their signaling pathways play a more critical role than epithelial cells in childhood and youth. The investigation of the TME in pediatric malignancies remains uncharted primarily, and this unique collection may help to include novel advances in this setting.

## Introduction

Pediatric cancer is diagnosed in more than 14,000 U.S. children (0–19 years) per year and in the twenty-first century, it remains the leading cause of disease-related death among children aged 1–14 years [1]. A few risk factors have been conclusively identified, including exposure to pesticides, high-dose radiation, and specific genetic syndromes, but the etiology underlying most events remains unknown. It is now well-recognized that neoplasms co-evolve with the tumor microenvironment (TME), which includes stromal cells, vasculature, fibroblasts, adipocytes, and different subsets of immunological cells. TME plays a crucial role in carcinogenesis, cancer formation, progression, dissemination, and resistance to therapy. Moreover, autophagy seems to be a vital regulator of the TME and controls tumor immunity. Autophagy is an evolutionarily conserved intracellular process. It enables the degradation and recycling of long-lived large molecules or damaged organelles using the lysosomal-mediated pathway. The multifaceted role of autophagy in the complicated neoplastic TME may depend on a specific context. Autophagy may function as a tumor-suppressive mechanism during early tumorigenesis by eliminating unhealthy intracellular components and proteins, regulating antigen presentation to and by immune cells, and supporting anti-cancer immune response. On the other hand, dysregulation of autophagy may contribute to tumor progression by promoting genome damage and instability. Finally, autophagy in TME stromal cells supplying nutrients to neoplastic cells may fuel growth in established neoplasms [2]. In this collection, we aim to gather contributions dealing with pediatric oncogenesis and papers targeting the role of autophagy and microenvironment in pediatric cancer. This perspective provides an assortment of regulatory substances that influence the features of the TME and the metastasis process. Mesenchymal cells in bone and soft-tissue sarcomas and their signaling pathways play a more critical role than epithelial cells in childhood and youth. The investigation of the TME in pediatric malignancies remains mostly uncharted. The highlighted discoveries primarily focus on genes that have been deregulated and are involved with processes such as cell adhesion, migration, and the spread of tumor cells. The involvement of these processes in developing a mesenchymal phenotype remains intriguing. It should be further explored, particularly regarding the epithelial-to-mesenchymal transition in epithelial malignancies. Various regulatory chemicals are impacting the characteristics of the TME and the process of metastasis [3, 4]. Priority is given to the signaling pathways that regulate the biology of mesenchymal cells in bone and soft-tissue malignant mesenchymal neoplasms (sarcomas) occurring in children and youth, as opposed to the epithelial cells, which are more commonly engaged in neoplasms in adults [5]. The exploration of the tumor microenvironment in pediatric cancers remains largely unexplored. The emphasized findings generally center around genes that have undergone deregulation and are implicated in processes such as cell adhesion, migration, and tumor cell dissemination. The role of these mechanisms in forming a mesenchymal phenotype and the spread of cancer is further investigated concerning the epithelial-to-mesenchymal transition (EMT) in epithelial tumors. The impact of cell plasticity on tumor activity is increasingly being acknowledged. Sarcomas are a diverse collection of tumors that present significant hurdles in terms of local recurrence and tumor dissemination, even with aggressive multimodal therapy. The ongoing research aims to identify and understand the molecular pathways responsible for spreading cancer cells to other body parts. Exploring the regulatory effects of structural components and non-malignant cell types that infiltrate the tumor is essential. Pediatric cancers often have a relatively low number of mutations, but they frequently have repeated chromosomal abnormalities [6]. Pathognomonic fusion genes are already being identified in the diagnostic routine for relevant sarcoma subtypes. Additionally, there is a correlation between the status of fusion genes and a poorer prognosis among non-metastatic patients [7]. Genetic and epigenetic alterations in tumor cells play a crucial role in tumor growth and can impact the tumor microenvironment.

Several studies have demonstrated how activated non-malignant mesenchymal cells of the stroma efficiently control matrix stiffness and display contractile and pro-invasive cell behavior [8] and, significantly, how these activities impact medication effectiveness and metastasis [9]. Nevertheless, much research on the tumor microenvironment has focused on epithelial entities that occur in maturity, such as breast carcinomas. Children’s mesenchymal tumors exhibit fundamental distinctions, and the role of non-neoplastic cells in these sarcomas is not well clarified. Without therapy, activated stromal cells are less prone to developing separate compartments, a characteristic commonly observed in epithelial malignancies. Alternatively, they mingle with tumor cells, immune cells, and other cell types within a pseudocapsule around the tumor or maybe aid in creating endothelial tubes during angiogenesis. The necessity of their structural support in advancing sarcoma has not been well examined, and the lack of distinct markers for non-malignant stromal cells in mesenchymal tumors adds complexity to these investigations. The regulation of metastatic dissemination of pediatric malignancies by the TME is still a developing area of investigation. Childhood sarcomas commonly consist of mesenchymal neoplasms, such as osteogenic sarcoma, primitive neuroectodermal tumor or Ewing sarcoma, and rhabdomyosarcoma. The prognosis of these neoplasms is generally favorable in comparison to numerous sarcomas in adults, while the issue of metastatic spread remains essential. Osteosarcoma primarily arises from skeletal tissue and is the tumorous subtype in which the neoplastic microenvironment has been extensively studied. Ewing sarcoma originates in either the bone or soft tissue. At the same time, rhabdomyosarcoma is a type of soft-tissue sarcoma that exhibits characteristics of skeletal muscle with evidence of a myogenic program.

## Sarcomas

Bone sarcomas typically exhibit pain, but soft-tissue sarcomas commonly present as painless masses. A detectable mass may or may not be existent in skeletal neoplasms. Constitutional symptoms, such as fatigue, weight loss, or widespread signs of inflammation accompanied by fever, are infrequently seen in cases where the tumors are sizable and have undergone necrosis [1]. Occasionally, the disease is transmitted upon diagnosis with clearly visible metastases. Rhabdomyosarcomas and Ewing sarcomas are classified as high-grade neoplasms, whereas osteosarcomas can exhibit high-grade or low-grade characteristics. The staging of tumors is determined using either the TNM system or the musculoskeletal society system. In the case of rhabdomyosarcoma, the grouping system based on clinical staging is used [10, 11].

Approximately 75–90% of juvenile sarcomas originate as a localized illness. However, it is expected that micrometastases are present in nearly all instances. This phenomenon is exemplified in the historical survival statistics of patients who did not undergo chemotherapy. In these cases, major surgery was found to be effective in controlling the cancer locally, but it was associated with a low overall survival rate [12]. The three primary categories of pediatric sarcomas exhibit a comparable pattern of metastasis, with hematogenous dissemination being the conventional pathway of propagation. The lungs are the primary site for metastasis, followed by the bones and bone marrow. Infrequently seen locations for rhabdomyosarcomas include lymph nodes, viscera, and soft tissues [13]. The prevalence of micrometastatic illness in high-grade pediatric sarcomas indicates that the mechanisms responsible for the spread of tumor cells are active at the initial phases of the disease. Contemporary treatment protocols involve administering systemic chemotherapy at an early stage to eliminate microscopic metastatic illness. This is done with the local removal of the primary tumor and extensive metastases, when possible. Neoadjuvant chemotherapy is commonly administered to patients to treat local illnesses. Following surgery, patients typically get further cycles of chemotherapy [14, 15]. Radiation therapy is obviously administered in patients exhibiting surgical margins, which are inadequate. Alternatively, it is used to locally control radiosensitive neoplasms, such as Ewing sarcomas and rhabdomyosarcomas, when the underlying tumor cannot be operated on. Identifying tumor-specific chromosomal translocations is often valuable for diagnosing pediatric mesenchymal neoplasms [16]. EWS-ETS gene fusion variations are present in Ewing sarcomas, while the predominant fusion genes linked to alveolar rhabdomyosarcoma are PAX3–FOXO1 and PAX7–FOXO1. The two primary subtypes of rhabdomyosarcoma, namely the alveolar subtype, which is more aggressive, can generally be differentiated using contemporary techniques, with a few exceptions. Despite embryonal rhabdomyosarcomas usually occurring sooner in the developing process than the alveolar subtype, they are still clinically and molecularly similar to fusion gene-negative alveolar rhabdomyosarcomas [17]. The outcome of pediatric sarcoma is contingent upon various criteria, such as the dimensions and location of the initial tumor, as well as the age of the patient [18]. The initial disease burden is of utmost importance in this setting, as children who have confined disease at presentation have a significantly more favorable prognosis compared to those with evident tumor dissemination. The primary determinant of patient outcome concerning treatment is the response to chemotherapy. In cases of bone sarcomas, the extent of necrosis following neoadjuvant treatment is commonly assessed [18, 19]. There are multiple histological methods available [1]. Individuals who do not respond well to treatment have a worse chance of recovering from cancer and are classified, based on commonly accepted standards, as those with less than 90% tumor necrosis caused by chemotherapy. Another significant aspect of treatment is the level of quality assurance reached after the examination of surgical margins [20].

## Cell migration and cell adhesion

Cell migration refers to the process by which cells move from one location to another within an organism. Metastatic dissemination, conversely, is the spread of cancer cells from the primary tumor to other parts of the body. Upon the initiation of metastasis, tumor cells embark on a complex series of steps, during which their ability to adapt to unfamiliar tissue microenvironments becomes crucial for survival. There are still numerous uncertainties regarding the selection mechanisms that occur throughout the evolution of diseases, specifically in cases when only specific sarcoma cells can reach distant organs and effectively metastasize. The subsequent conversation centers around the flexibility of mesenchymal cells and the cell adhesion molecules that play a role in cell migration and metastasis. It is worth mentioning that the characteristics of mesenchymal qualities in sarcoma are controlled by various signaling pathways involved in development. A recent study conducted by others has discussed some of these pathways [21]. Cell migration can be classified into two main types: collective cell migration, which occurs in epithelial malignancies, and individual cell migration, which happens in sarcoma. Sarcoma mesenchymal cell migration can occur individually or in chains. It is generally controlled by the extracellular matrix (ECM), where different integrins and proteases play major roles. Cadherins, which create adherens junctions, significantly facilitate direct cell–cell interactions in multicellular organisms. Mesenchymal adherens junctions are anticipated to have a shorter duration than the epithelial equivalent, and their stability is partially governed by endocytosis and modulation of the cytoskeleton. Downregulation of E-cadherin is a crucial step in the cellular process of epithelial-to-mesenchymal transition (EMT). At the same time, its overexpression is associated with the mesenchymal to epithelial transition (MET) during the formation of distant metastasis. Osteosarcoma has been observed to undergo a mesenchymal to amoeboid transition (MAT) while migrating via endothelial cells [22]. A recent assessment of mesenchymal features in epithelial tumors found that a partial EMT was uncovered to be profitable for the tumor-initiating ability. However, drug resistance reached a plateau and remained constant when the EMT program was further activated [23]. The effectiveness of invasiveness was highest when there was a robust activation of EMT, resulting in the migration of individual cells rather than the typical migration of multicellular carcinoma cells. The process of EMT in sarcoma is inherently less apparent. However, it is established that the expression of E-cadherin also contributes to the inhibition of anchorage-independent growth and spheroid formation [24]. Claudin-1, a protein that forms tight junctions, is an epithelial differentiation marker that can be identified in sarcoma [25] as well as other epithelial differentiation markers, such keratins [26–32]. It has been demonstrated that epithelial markers in sarcomas are associated with a better prognosis for patients as identified by the meta-analysis of Wang et al. [33]. It may seem counterintuitive, but research has demonstrated that the deliberate activation of mesenchymal-associated adhesion molecules can hinder the movement and spread of cells in osteosarcoma while promoting bone metastasis in osteosarcoma and Ewing sarcoma [34, 35]. Nevertheless, typical osteoblasts exhibit a significant expression of cadherin-11 and N-cadherin, which are crucial in regulating cell function and differentiation. Hence, a TME specific to a particular subtype may elucidate why decreased levels are believed to play a significant role in advancing osteosarcoma and its spread to other parts of the body [36]. Cadherin switching and activating N-cadherin, an EMT marker, are linked to transforming malignant cells derived from epithelial tissues into a mesenchymal phenotype. This transformation is characterized by alterations in cell morphology and the acquisition of migratory and invasive capabilities. Analogous pathways have also been documented in mesenchymal cancers. For instance, activating N-cadherin and alpha9-integrin promotes the invasion of cells in rhabdomyosarcoma through a mechanism that relies on Notch signaling [37]. The Notch signaling pathway is a developmental mechanism that has a role in sarcoma growth by regulating cell motility, stemness, and angiogenesis. Endothelial cells and pericytes have been proposed as potential sources for activating Notch in osteosarcoma [38]. Sirtuin 1 (SIRT1) acts as a key regulator of vascular endothelial homeostasis, angiogenesis, and endothelial dysfunction and SIRT1 acts as an intrinsic negative modulator of Notch signaling in endothelial cells, which may be at the basis of the osteoclastogenesis vs. osteoblastogenesis in osteosarcoma [39, 40]. Crucially, it is widely considered that unregulated developmental processes have a significant impact on juvenile sarcomas. Currently, several published articles discuss the roles of epithelial and mesenchymal tissue markers in setting cell migrations in pediatric sarcoma. Preussner et al. examined the significance of epithelial/mesenchymal states regarding tumor cell plasticity in a hereditary rodent model (mouse) of rhabdomyosarcoma [41]. Within a genetically unstable and susceptible milieu of regenerated muscle, muscle stem cells triggered the formation of tumors through a process like the MET pathway, facilitated by the activation of zygotic Dux transcription factors. When Duxbl was excessively expressed in normal muscle stem cells during the experiment, it led to the expression of cadherin, the ability to become immortal, and the capability to develop tumors. The authors additionally established a connection between Dux transcription factors and the expression profiles of stem cells in malignancies originating from germ cells or epithelial cells supporting the existence of tumor heterogeneity and the identification of stem cell characteristics in rhabdomyosarcoma.

## Dissemination models

The invasive characteristics of primary tumors may not necessarily indicate the ability to form distant metastases, which can lead to reduced overall survival. A recent study conducted by EpSSG revealed that tiny pulmonary nodules may be present in more than 20% of localized rhabdomyosarcoma cases at the time of diagnosis. However, it was not observed that these nodules impacted survival rates [42]. It is crucial to comprehend the factors that govern the proliferation of metastatic cells before, during, or after treatment, both locally and systemically, at various levels. The conventional linear progression model of metastasis is founded on the premise that genetic changes gradually accumulate inside the tumor, leading to the acquisition of metastatic characteristics by subclonal populations. The available evidence strongly supports an early and efficient spread of the phenomenon, together with the occurrence of concurrent advancements and colonization routes [43]. This approach aligns with the notion that the genetic changes occurring at metastatic sites can differ significantly from those observed in the parent tumor. Irrespective of the timing of dissemination in tumor progression, it also entails metastatic expansion in various anatomical sites particular to the type of tumor. The organotropic model of metastasis explains how the preference of certain tumor cells (seed) for specific organs (soil) enables successful metastasis. This model builds upon the traditional seed and soil theory but with modifications considering organ tropism’s role and pre-metastatic habitats [44].

On the other hand, the anatomical/mechanical model thinks of metastatic clones being filtered and flowing, with anatomical obstacles regulating their spread [44]. The extent to which each model contributes to different tumor types may be debatable. Still, it is evident that the presence of circulating tumor cells is a common occurrence, and the process of metastasis is widely regarded as inefficient [44]. The sarcoma tumor microenvironment exhibits significant variability based on subtype, anatomical location, age, gender, genetic complexity, and previous treatment. Various sources have recently examined a comprehensive analysis of the significance of vascular cells, immune cells, and immunotherapy in sarcoma [45, 46].

## Extracellular matrix and its associated proteins

Weaver and other researchers have made persuasive contributions that have enhanced our fundamental comprehension of the significance of matrix stiffness and physical surroundings concerning tumor growth [47]. Sarcomas exhibit molecular results indicating that the physical (bio-mechanical) and chemical features of the TME synergistically contribute to the enhancement of sarcoma motility and metastasis through a feedback loop [48]. Nevertheless, ECM proteins frequently exert multiple effects in the TME and should be evaluated context-dependently concerning the specific organ and tissue involved. The TME consists of malignant, non-malignant stromal, vascular, and immune cells. Collagens, structural ECM proteins, and osteopontin, a matricellular protein, are crucial in providing both physical support and signaling cues essential for cell movements. Transforming growth factor-beta (TGFβ) regulates many proteins involved with the ECM, and these proteins have the potential to serve as biomarkers [49]. Sarcomas possess a distinctive characteristic where the differentiation between neoplastic cells and mesenchymal cells is remarkably ambiguous, primarily because of the stromal origin of the neoplastic cells. Cellular transdifferentiation of mesenchymal stem cells produced from bone marrow can also take place, and this process is recognized explicitly as significant in the advancement of osteosarcoma [22]. The presence of low oxygen levels in a TME usually promotes the growth of the tumor. The effects of varying oxygen levels within the tumor on the invasion of sarcoma cells have been investigated [50]. In humans, the *EPAS1* gene is responsible for coding EPAS1 protein, an alias of which is HIF2alpha, an acronym for hypoxia-inducible factor 2 alpha. EPAS1 is a type of hypoxia-inducible factors, which are collected as a group of transcription factors involved in body response to oxygen level [51]. The vascular endothelial growth factor (VEGF) promoter, particularly in situations of hypoxia, is a downstream target of HIF-2 (other than HIF-1), and the expression levels of either HIF-1α or HIF-2α correlate positively to VEGF expression. In the future, there may be incitement to further evaluate protein–protein interaction and using experimental animal models [51]. HIF1α triggers the activation of the SDF1–CXCR4 signaling pathway in response to hypoxia [51]. Notably, the increased levels of the chemokine receptor CXCR4 remain present even when cells are exposed to normal oxygen levels again [52]. Sarcoma research has provided evidence that the SDF-1 ligand probably stimulates chemotaxis through membranes. This ligand may also act into the adherence to endothelial cells, and the production of matrix metalloproteinase 2 (MMP-2) [53]. There have been studies emphasizing that MMP-2 and MMP-9 may be considered prognostic indicators and are linked to the spread of cancer to other body parts in osteosarcoma [54–57]. The display of CXCR4 is seen in two-thirds of osteosarcomas and is associated with the expression of VEGF and reduced survival rates in patients [58, 59]. Rhabdomyosarcoma has also been associated with a link indicating reduced patient survival [60]. Currently, there are many tumor environments in which CXCR4-positive cancer cells are highly likely to spread to tissues that express SDF1 (CXCL12), such as the bone marrow [53]. The characteristic bone marrow milieu, containing both resident stem cells and progenitor cells, has a natural inclination to attract and sustain propagating tumor cells from many sources. Additional research confirms that the direct engagement or enlistment of mesenchymal stem cells originating from bone marrow enhances the growth and infiltration of the primary tumor [61]. One suggested way mesenchymal stem cells may function in the tumor microenvironment is by promoting stemness and resistance to chemotherapy through the NFκB pathway and release of IL6 [62]. Lysyl oxidases (LOX), which are recognized as potent regulators of structural alterations in healthy connective tissue, fibrotic conditions, and cancer, are critical in the process of tumor seeding [63]. The LOX family comprises catalytic enzymes that form cross-links between collagen and elastin within the tumor microenvironment. Multiple studies have now shown that LOX family members play an active role in the advancement of tumors and the spread of cancer to other parts of the body, regardless of the kind of tumor. Furthermore, there are documented accounts of tumor-suppressive effects, specifically in osteosarcoma [64]. The EWS-FLI oncoprotein in Ewing sarcoma decreases the expression of LOX, and the observed tumor-suppressive effects have been associated with a specific pro-peptide domain [65]. LOX and LOXL1 both possess pro-domains and undergo extracellular processing, distinguishing them from the family members, namely LOXL2, LOXL3, and LOXL4. Activating the mature protein necessitates the proteolytic elimination of its N-terminal LOX-propeptide, also known as LOX-PP. Thrombospondin-1 (TSP1) is a well-known glycoprotein found in the TME. It is mostly known for its ability to inhibit the formation of new blood vessels (anti-angiogenic) and its influence on the invasion of tumor cells. TSP1 achieves these effects by interacting with several molecules on the cell surface and matrix metalloproteinases [66, 67]. The α4β1 integrin has been associated with the pro-adhesive actions in osteosarcoma [68]. Since the approval of trabectedin for the treatment of advanced or metastatic soft-tissue sarcoma, various pharmacological mechanisms of action have been suggested, including anti-angiogenic effects on the cells of the endothelium and the elevation of TSP1 [69]. The study demonstrated that the TME produced higher levels of tissue inhibitor of metalloproteinases 1 and 2, leading to poor extracellular matrix remodeling. The extent to which TSP1 can function as a regulator of angiogenesis-dependent “dormancy” is yet unidentified.

## Growth factors

Cell proliferation and differentiation in mesenchymal stem cells are frequently interconnected and controlled by growth factors such as TGFβ, platelet-derived growth factor (PDGF), and fibroblast growth factor (FGF) [70]. TGFβ is recognized as a crucial controller of the EMT phenomenon and the corresponding advancement of tumors in various types of cancer. High levels of TGFβ in osteosarcoma are associated with the grade of the tumor, resistance to chemotherapy, and the presence of metastases [68, 69]. Likewise, when the EMT transcription factors Snails, ZEBs, or Twist are excessively produced, it encourages the spreading of tumor cells [71]. Conversely, when the inhibitory transcription factor Smad7 is excessively produced or when illness development is prevented through pharmacological means, it hinders the advancement of the disease [72–76]. Nevertheless, genetic modifications and in vivo analyses also reveal the ability of TGFβ signaling in sarcoma to limit tumor growth [77]. TGFβ family members affect several cell types inside the TME. The TGFβ co-receptor endoglin is recognized as a marker for blood vessels in tumor biology. However, it is also seen in malignant cells and has been associated with tumor cell plasticity and poorer patient survival in Ewing sarcoma [78]. Additional factors involved in TGFβ-controlling angiogenesis include VEGF and connective tissue growth factor (CTGF) [79]. The appearance of VEGF has been linked to the density of blood vessels and a reduced period of disease-free survival in patients with osteosarcoma [80–82]. Recent studies have demonstrated that CTGF stimulates the formation of new blood vessels, enhances the production of MMP-2/3, and promotes the movement of cells in osteosarcoma. Conversely, reducing the levels of CTGF in an experimental mouse model decreased the spread of cancer to the lungs [81–84]. Additional research has demonstrated that CTGF can enhance osteosarcoma's resistance to drugs and control VEGF production from fibroblasts [85, 86]. The TGFβ pathway is also involved in the specific inhibition of the immunologic system [87, 88]. Osteosarcoma cells can control the recruitment and differentiation of infiltrating immune cells and build a localized immunologically tolerant milieu, hence facilitating tumor growth [89]. Additional experiments have demonstrated that the immune response in osteosarcoma can be reinstated by pairing an anti-TGFβ antibody with dendritic cells [90]. Gao et al. recently elucidated a novel method via which cancers evade detection by the innate immune system. They employed a model system of methylcholanthrene (MCA)-induced fibrosarcoma to investigate this phenomenon. The study proposed that tumor immunoevasion induced by TGFβ involved the transformation of anti-tumoral NK cells into type 1 innate lymphoid cells, resulting in the loss of their capacity to regulate local tumor growth and metastasis [91]. The PDGF pathway is a developmental signaling route that may be triggered during the formation of sarcomas. A recent study demonstrated that PDGF signaling maintains cancer stem cell characteristics, including self-renewal, tissue invasion, and resistance to chemotoxic therapy, in sarcoma [92, 93]. Increased levels of phosphorylated PDGFRα/β and EMT proteins were noted in spheroid cultures, which are enhanced for neoplastic stem cells. However, the migration and invasion were significantly reduced by up to 80% when treated with the tyrosine kinase inhibitor imatinib, which targets PDGFRα/β. Additionally, the expression of EMT proteins was also reduced. These findings align with previously documented oncogenic mechanisms of PDGF signaling, such as the self-stimulation of tumor cells, stimulation of surrounding mesenchymal cells of the stroma, promotion of blood vessel growth, and direction of tumor interstitial fluid pressure (IFP), which affects the movement of substances in and out of the tumor [94, 95]. Overall, the PDGF family is associated with initial tumor growth, metastasis, medication resistance, and unfavorable clinical outcomes in various types of cancers. However, the specific impact of PDGF activity on different subtypes of sarcoma is still not well understood [96]. PDGF receptor genetic abnormalities are found in around 2% of pediatric malignancies [6]. However, PDGF ligands and/or receptors are commonly found in osteosarcoma, PNET/Ewing sarcoma, and rhabdomyosarcoma, and there is a connection between their presence and the patient’s clinical fate [1, 5, 45, 96–99]. Notably, fusion genes such as PAX3–FOXO1 (found in alveolar rhabdomyosarcoma) and EWS-ETS (found in primitive neuroectodermal tumor or Ewing sarcoma) can intentionally trigger the expression of PDGF family members through experimental means [98, 99]. Significantly, the resistance mechanisms to treatment drugs in sarcoma have been found to include disrupted PDGF signaling. An illustrative instance of this phenomenon is the documented reciprocal influence between CXCR4 and PDGF signaling in Ewing sarcoma, wherein heightened CXCR4 expression is associated with metastasis and unfavorable patient prognosis [100, 101]. Upon administration of a CXCR4-targeting drug to tumor cells, activating PDGFRβ as a compensatory mechanism enhanced cell proliferation. However, applying a multi-kinase inhibitor, specifically dasatinib, effectively countered this effect. A recent study on rhabdomyosarcoma has discovered that the overexpression and constant activation of PDGFRα, along with its amplification, serve as a mechanism of acquired resistance to a drug that aims the insulin-like growth factor I receptor (IGF-IR) [102]. Collectively, these results emphasize the necessity of exploring the mechanisms by which anti-cancer drugs work to identify appropriate treatment combinations.

## Most recent breaking update and future investigations

Metastasis is driven by a detailed and thorough cooperation between a neoplasm and its microenvironment, which results in the adaptation of molecular mechanisms that evade the immune system. It enables pre-metastatic niche formation. Roberts et al. studied the interferon regulatory factor 5 (IRF5) and found that its expression in osteosarcoma clinically correlates with prolonged survival and decreased secretion of tumor-derived extracellular vesicles (t-dEVs). The packaging of IRF5 mRNA in EVs caused downstream effects of decrease in the metastatic burden and an anti-tumorigenic microenvironment [103]. Phosphodiesterase 1B (PDE1B) has also been considered a potential biomarker associated with TME and clinical significance in osteosarcoma [104]. PDE1B high expression was related to a better tumor prognosis, suppressing immune escape from osteosarcoma [104]. Long noncoding RNA (lncRNA) is a non-coding RNA. LncRNAs have a length of more than 200 nucleotides and are involved in multiple regulatory processes in vivo, and pathology of several human diseases [105]. ROR1-AS1 is a cancer-associated lncRNA. This lncRNA is either over- or underexpressed in multiple malignancies, including colon carcinoma, hepatocellular carcinoma, and osteosarcoma among others [106, 107]. WNT5B (WNT Family Member 5B) expression has been identified as high in osteosarcoma stem cells [108]. It leads to increased stem cell proliferation and migration through the stemness gene SOX2 (SRY-box transcription factor 2).

Historically, the treatment of pediatric sarcomas has predominantly focused on chemotherapy. Different medicines have been employed, all of which share the characteristic of selectively killing malignant cells in both the primary tumor and any metastatic locations. We are transitioning into a new phase in the field of oncology, primarily defined using combination therapies and targeted treatments that specifically target cancerous cells and/or cells within the TME. Notable instances include the use of imatinib to treat dermatofibrosarcoma protuberans and gastrointestinal stromal tumors, as well as the use of pazopanib to treat metastatic non-adipocytic soft-tissue sarcoma [109]. Trabectedin, an alternative treatment for sarcoma, has received approval from the European Medicines Agency explicitly for curing soft-tissue sarcomas in adults. Trabectedin not only directly affects malignant cells but also alters the characteristics of tumor-associated macrophages. Muramyl tripeptide (mifamurtide) is a European Medicines Agency-approved therapeutic regimen targeting macrophages. It is utilized for the treatment of osteosarcoma. Immunotherapy is a new and promising approach to regulate the activity of immune cells in certain groups of patients. However, its use in sarcoma is still in the early stages of research. Investigations into the manipulation of the immune response in the microenvironment of pediatric sarcoma are also conducted through the utilization of tumor vaccines. The efficacy of such treatments is yet to be determined.

## Conclusive remarks

**Fig. 1:**
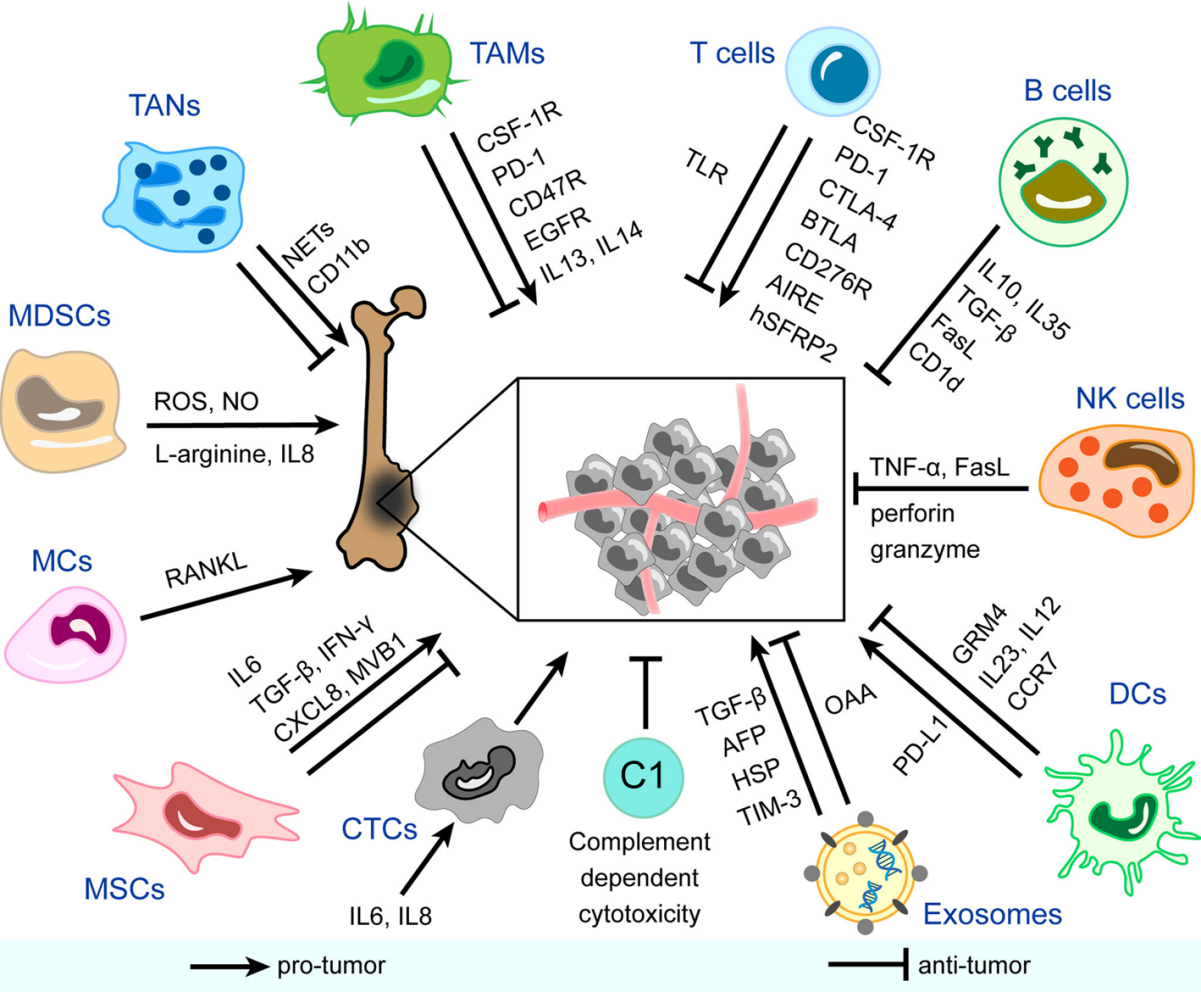
Immune and non-immune components in the immune microenvironment of osteosarcoma and mechanisms of their pro-tumor/anti-tumor effects. *CSF-1R* colony-stimulating factor 1 receptor, *PD-1* programmed cell death protein-1, *EGFR* epidermal growth factor receptor, *IL* interleukin, *NETs* neutrophil extracellular traps, *ROS* reactive oxygen species, *NO* nitric oxide, *RANKL* receptor activator NF-κB ligand, *TGF-β* transforming growth factor-beta, *IFN-γ* interferon-gamma, *CXCL8* C-X-C motif chemokine ligand 8, *AFP* α-fetoprotein, *HSP* heat shock protein, *TIM-3* T cell immunoglobulin and mucin domain-containing protein-3, *OAA* osteosarcoma-associated antigens, *PD-L1* programmed cell death protein ligand-1, *GRM4* glutamate metabotropic receptor 4, *CCR7* chemokine receptor 7, *TNF-α* tumor necrosis factor-alpha, *CTLA-4* cytotoxic T-lymphocyte-associated protein-4, *BTLA* B And T-lymphocyte attenuator, *AIRE* autoimmune regulator expression, *hSFRP2* humanized secreted frizzled-related protein 2, *TLR* toll-like receptor, *TAMs* tumor-associated macrophages, *TANs* tumor-associated neutrophils, *MDSCs* myeloid-derived suppressor cells, *MCs* mast cells, *MSCs* mesenchymal stem cells, *CTCs* circulating tumor cells, *C* complement, *DCs* dendritic cells, *NK cells* natural killer cells (Source: Zhu et al. [110])

Gaining a deeper comprehension of the activities occurring in the TME throughout pediatric sarcoma is crucial for enhancing patient prognosis and quality of life. Research on prevalent epithelial malignancies has been valuable in pinpointing potential molecular pathways implicated in the spread of cancer cells and resistance to treatment in sarcoma, specifically osteosarcoma (Fig. 1). Nevertheless, sarcomas possess a distinct mesenchymal origin, which sets them apart from epithelial tumors and necessitates a different approach when examining cellular processes such as EMT and MET. The diversity among different sarcoma subtypes, as well as within each subtype, poses significant difficulties. Therefore, applying discoveries from other contexts to pediatric sarcoma requires additional investigation.

## Data Availability

No datasets were generated or analysed during the current study.

## References

[1] Sergi CM. Pathology of childhood and adolescence. Springer; 2020.

[2] Klionsky DJ, Abdel-Aziz AK, Abdelfatah S, Abdellatif M, Abdoli A, Abel S, Abeliovich H, Abildgaard MH, Abudu YP, Acevedo-Arozena A, et al. Guidelines for the use and interpretation of assays for monitoring autophagy (4th edition)(1). Autophagy. 2021;17:1–382. 10.1080/15548627.2020.1797280. 33634751 10.1080/15548627.2020.1797280 PMC7996087

[3] Ribatti D, Tamma R, Annese T. Epithelial-mesenchymal transition in cancer: a historical overview. Transl Oncol. 2020;13: 100773. 10.1016/j.tranon.2020.100773. 32334405 10.1016/j.tranon.2020.100773 PMC7182759

[4] Ribatti D. Epithelial-mesenchymal transition in morphogenesis, cancer progression and angiogenesis. Exp Cell Res. 2017;353:1–5. 10.1016/j.yexcr.2017.02.041. 28257786 10.1016/j.yexcr.2017.02.041

[5] Ehnman M, Chaabane W, Haglund F, Tsagkozis P. The tumor microenvironment of pediatric sarcoma: mesenchymal mechanisms regulating cell migration and metastasis. Curr Oncol Rep. 2019;21:90. 10.1007/s11912-019-0839-6. 31418125 10.1007/s11912-019-0839-6 PMC6695368

[6] Chmielecki J, Bailey M, He J, Elvin J, Vergilio JA, Ramkissoon S, Suh J, Frampton GM, Sun JX, Morley S, et al. Genomic profiling of a large set of diverse pediatric cancers identifies known and novel mutations across tumor spectra. Can Res. 2017;77:509–19. 10.1158/0008-5472.Can-16-1106. 10.1158/0008-5472.Can-16-1106 PMC8191391 28069802

[7] Missiaglia E, Williamson D, Chisholm J, Wirapati P, Pierron G, Petel F, Concordet JP, Thway K, Oberlin O, Pritchard-Jones K, et al. PAX3/FOXO1 fusion gene status is the key prognostic molecular marker in rhabdomyosarcoma and significantly improves current risk stratification. J Clin Oncol. 2012;30:1670–7. 10.1200/JCO.2011.38.5591. 22454413 10.1200/JCO.2011.38.5591

[8] Foster CT, Gualdrini F, Treisman R. Mutual dependence of the MRTF-SRF and YAP-TEAD pathways in cancer-associated fibroblasts is indirect and mediated by cytoskeletal dynamics. Genes Dev. 2017;31:2361–75. 10.1101/gad.304501.117. 29317486 10.1101/gad.304501.117 PMC5795783

[9] Miller BW, Morton JP, Pinese M, Saturno G, Jamieson NB, McGhee E, Timpson P, Leach J, McGarry L, Shanks E, et al. Targeting the LOX/hypoxia axis reverses many of the features that make pancreatic cancer deadly: inhibition of LOX abrogates metastasis and enhances drug efficacy. EMBO Mol Med. 2015;7:1063–76. 10.15252/emmm.201404827. 26077591 10.15252/emmm.201404827 PMC4551344

[10] Fletcher CD. The evolving classification of soft tissue tumours—an update based on the new 2013 WHO classification. Histopathology. 2014;64:2–11. 10.1111/his.12267. 24164390 10.1111/his.12267

[11] Dasgupta R, Fuchs J, Rodeberg D. Rhabdomyosarcoma. Semin Pediatr Surg. 2016;25:276–83. 10.1053/j.sempedsurg.2016.09.011. 27955730 10.1053/j.sempedsurg.2016.09.011

[12] Jaffe N, Puri A, Gelderblom H. Osteosarcoma: evolution of treatment paradigms. Sarcoma. 2013;2013: 203531. 10.1155/2013/203531. 23781130 10.1155/2013/203531 PMC3678494

[13] Nakamura T, Matsumine A, Matsubara T, Asamuma K, Niimi R, Uchida A, Sudo A. Retrospective analysis of metastatic sarcoma patients. Oncol Lett. 2011;2:315–8. 10.3892/ol.2011.238. 22866083 10.3892/ol.2011.238 PMC3410588

[14] Marina NM, Smeland S, Bielack SS, Bernstein M, Jovic G, Krailo MD, Hook JM, Arndt C, van den Berg H, Brennan B, et al. Comparison of MAPIE versus MAP in patients with a poor response to preoperative chemotherapy for newly diagnosed high-grade osteosarcoma (EURAMOS-1): an open-label, international, randomised controlled trial. Lancet Oncol. 2016;17:1396–408. 10.1016/s1470-2045(16)30214-5. 27569442 10.1016/s1470-2045(16)30214-5 PMC5052459

[15] Werier J, Yao X, Caudrelier JM, Di Primio G, Ghert M, Gupta AA, Kandel R, Verma S. A systematic review of optimal treatment strategies for localized Ewing’s sarcoma of bone after neo-adjuvant chemotherapy. Surg Oncol. 2016;25:16–23. 10.1016/j.suronc.2015.11.002. 26979636 10.1016/j.suronc.2015.11.002

[16] Chang CC, Shidham VB. Molecular genetics of pediatric soft tissue tumors: clinical application. J Mol Diagn. 2003;5:143–54. 10.1016/s1525-1578(10)60466-7. 12876204 10.1016/s1525-1578(10)60466-7 PMC1907327

[17] Stewart E, McEvoy J, Wang H, Chen X, Honnell V, Ocarz M, Gordon B, Dapper J, Blankenship K, Yang Y, et al. Identification of therapeutic targets in rhabdomyosarcoma through integrated genomic, epigenomic, and proteomic analyses. Cancer Cell. 2018;34:411-426.e419. 10.1016/j.ccell.2018.07.012. 30146332 10.1016/j.ccell.2018.07.012 PMC6158019

[18] Smeland S, Bielack SS, Whelan J, Bernstein M, Hogendoorn P, Krailo MD, Gorlick R, Janeway KA, Ingleby FC, Anninga J, et al. Survival and prognosis with osteosarcoma: outcomes in more than 2000 patients in the EURAMOS-1 (European and American Osteosarcoma Study) cohort. Eur J Cancer. 2019;109:36–50. 10.1016/j.ejca.2018.11.027. 30685685 10.1016/j.ejca.2018.11.027 PMC6506906

[19] Bacci G, Ferrari S, Bertoni F, Rimondini S, Longhi A, Bacchini P, Forni C, Manfrini M, Donati D, Picci P. Prognostic factors in nonmetastatic Ewing’s sarcoma of bone treated with adjuvant chemotherapy: analysis of 359 patients at the Istituto Ortopedico Rizzoli. J Clin Oncol. 2000;18:4–11. 10.1200/jco.2000.18.1.4. 10623687 10.1200/jco.2000.18.1.4

[20] Jeys LM, Thorne CJ, Parry M, Gaston CL, Sumathi VP, Grimer JR. A novel system for the surgical staging of primary high-grade osteosarcoma: the birmingham classification. Clin Orthop Relat Res. 2017;475:842–50. 10.1007/s11999-016-4851-y. 27138473 10.1007/s11999-016-4851-y PMC5289182

[21] Deel MD, Li JJ, Crose LE, Linardic CM. A review: molecular aberrations within hippo signaling in bone and soft-tissue sarcomas. Front Oncol. 2015;5:190. 10.3389/fonc.2015.00190. 26389076 10.3389/fonc.2015.00190 PMC4557106

[22] Pietrovito L, Leo A, Gori V, Lulli M, Parri M, Becherucci V, Piccini L, Bambi F, Taddei ML, Chiarugi P. Bone marrow-derived mesenchymal stem cells promote invasiveness and transendothelial migration of osteosarcoma cells via a mesenchymal to amoeboid transition. Mol Oncol. 2018;12:659–76. 10.1002/1878-0261.12189. 29517849 10.1002/1878-0261.12189 PMC5928379

[23] Shibue T, Weinberg RA. EMT, CSCs, and drug resistance: the mechanistic link and clinical implications. Nat Rev Clin Oncol. 2017;14:611–29. 10.1038/nrclinonc.2017.44. 28397828 10.1038/nrclinonc.2017.44 PMC5720366

[24] Jolly MK, Ware KE, Xu S, Gilja S, Shetler S, Yang Y, Wang X, Austin RG, Runyambo D, Hish AJ, et al. E-Cadherin represses anchorage-independent growth in sarcomas through both signaling and mechanical mechanisms. Mol Cancer Res. 2019;17:1391–402. 10.1158/1541-7786.Mcr-18-0763. 30862685 10.1158/1541-7786.Mcr-18-0763 PMC6548594

[25] Schuetz AN, Rubin BP, Goldblum JR, Shehata B, Weiss SW, Liu W, Wick MR, Folpe AL. Intercellular junctions in Ewing sarcoma/primitive neuroectodermal tumor: additional evidence of epithelial differentiation. Mod Pathol. 2005;18:1403–10. 10.1038/modpathol.3800435. 15920547 10.1038/modpathol.3800435

[26] Cheng Y, Bai Q, Wu B, Chang B, Bi R, Yang W, Wang J, Tu X. Clinicopathologic and molecular cytogenetic analysis of 8 cases with uterine cervical Ewing sarcoma: case series with literature review. Am J Surg Pathol. 2021;45:523–30. 10.1097/PAS.0000000000001674. 33538423 10.1097/PAS.0000000000001674

[27] Creytens D, Ferdinande L, Van Dorpe J. Multifocal cytokeratin expression in a dedifferentiated solitary fibrous tumor with heterologous rhabdomyosarcomatous differentiation: a challenging diagnosis! Int J Surg Pathol. 2018;26:423–7. 10.1177/1066896918758452. 29482421 10.1177/1066896918758452

[28] Elbashier SH, Nazarina AR, Looi LM. Cytokeratin immunoreactivity in Ewing sarcoma/primitive neuroectodermal tumour. Malays J Pathol. 2013;35:139–45. 24362477

[29] Greco MA, Steiner GC, Fazzini E. Ewing’s sarcoma with epithelial differentiation: fine structural and immunocytochemical study. Ultrastruct Pathol. 1988;12:317–25. 10.3109/01913128809098044. 2456637 10.3109/01913128809098044

[30] Li Q, Cui W, Abulajiang G, Ma Y, Liu X, Zhang W, Li X. Application of immunohistochemistry in the diagnosis of small round blue-cell tumors of soft tissue. Clin Lab. 2014;60:1383–92. 10.7754/clin.lab.2013.130909. 25185426 10.7754/clin.lab.2013.130909

[31] Machado I, Noguera R, Santonja N, Donat J, Fernandez-Delgado R, Acevedo A, Baragano M, Navarro S. Immunohistochemical study as a tool in differential diagnosis of pediatric malignant rhabdoid tumor. Appl Immunohistochem Mol Morphol. 2010;18:150–8. 10.1097/PAI.0b013e3181b91a51. 19770707 10.1097/PAI.0b013e3181b91a51

[32] Srivastava A, Rosenberg AE, Selig M, Rubin BP, Nielsen GP. Keratin-positive Ewing’s sarcoma: an ultrastructural study of 12 cases. Int J Surg Pathol. 2005;13:43–50. 10.1177/106689690501300106. 15735854 10.1177/106689690501300106

[33] Wang N, He YL, Pang LJ, Zou H, Liu CX, Zhao J, Hu JM, Zhang WJ, Qi Y, Li F. Down-regulated E-cadherin expression is associated with poor five-year overall survival in bone and soft tissue sarcoma: results of a meta-analysis. PLoS ONE. 2015;10: e0121448. 10.1371/journal.pone.0121448. 25822802 10.1371/journal.pone.0121448 PMC4378985

[34] Kashima T, Nakamura K, Kawaguchi J, Takanashi M, Ishida T, Aburatani H, Kudo A, Fukayama M, Grigoriadis AE. Overexpression of cadherins suppresses pulmonary metastasis of osteosarcoma in vivo. Int J Cancer. 2003;104:147–54. 10.1002/ijc.10931. 12569568 10.1002/ijc.10931

[35] Hatano M, Matsumoto Y, Fukushi J, Matsunobu T, Endo M, Okada S, Iura K, Kamura S, Fujiwara T, Iida K, et al. Cadherin-11 regulates the metastasis of Ewing sarcoma cells to bone. Clin Exp Metastasis. 2015;32:579–91. 10.1007/s10585-015-9729-y. 26092671 10.1007/s10585-015-9729-y

[36] Nakajima G, Patino-Garcia A, Bruheim S, Xi Y, San Julian M, Lecanda F, Sierrasesumaga L, Müller C, Fodstad O, Ju J. CDH11 expression is associated with survival in patients with osteosarcoma. Cancer Genomics Proteomics. 2008;5:37–42. 18359978

[37] Masià A, Almazán-Moga A, Velasco P, Reventós J, Torán N, Sánchez de Toledo J, Roma J, Gallego S. Notch-mediated induction of N-cadherin and α9-integrin confers higher invasive phenotype on rhabdomyosarcoma cells. Br J Cancer. 2012;107:1374–83. 10.1038/bjc.2012.411. 22976797 10.1038/bjc.2012.411 PMC3494428

[38] McManus MM, Weiss KR, Hughes DP. Understanding the role of Notch in osteosarcoma. Adv Exp Med Biol. 2014;804:67–92. 10.1007/978-3-319-04843-7_4. 24924169 10.1007/978-3-319-04843-7_4

[39] Sergi C, Shen F, Liu SM. Insulin/IGF-1R, SIRT1, and FOXOs pathways-an intriguing interaction platform for bone and osteosarcoma. Front Endocrinol (Lausanne). 2019;10:93. 10.3389/fendo.2019.00093. 30881341 10.3389/fendo.2019.00093 PMC6405434

[40] Guarani V, Deflorian G, Franco CA, Kruger M, Phng LK, Bentley K, Toussaint L, Dequiedt F, Mostoslavsky R, Schmidt MHH, et al. Acetylation-dependent regulation of endothelial Notch signalling by the SIRT1 deacetylase. Nature. 2011;473:234–8. 10.1038/nature09917. 21499261 10.1038/nature09917 PMC4598045

[41] Preussner J, Zhong J, Sreenivasan K, Günther S, Engleitner T, Künne C, Glatzel M, Rad R, Looso M, Braun T, et al. Oncogenic amplification of zygotic dux factors in regenerating p53-deficient muscle stem cells defines a molecular cancer subtype. Cell Stem Cell. 2018;23:794-805.e794. 10.1016/j.stem.2018.10.011. 30449715 10.1016/j.stem.2018.10.011

[42] Vaarwerk B, Bisogno G, McHugh K, Brisse HJ, Morosi C, Corradini N, Jenney M, Orbach D, Chisholm JC, Ferrari A, et al. Indeterminate Pulmonary nodules at diagnosis in rhabdomyosarcoma: are they clinically significant? A report from the European paediatric soft tissue sarcoma study group. J Clin Oncol. 2019;37:723–30. 10.1200/jco.18.01535. 30702969 10.1200/jco.18.01535

[43] Ghajar CM, Bissell MJ. Metastasis: pathways of parallel progression. Nature. 2016;540:528–9. 10.1038/nature21104. 27974802 10.1038/nature21104

[44] Paget S. The distribution of secondary growths in cancer of the breast. 1889. Cancer Metastasis Rev. 1989;8:98–101. 2673568

[45] Ehnman M, Larsson O. microenvironmental targets in sarcoma. Front Oncol. 2015;5:248. 10.3389/fonc.2015.00248. 26583076 10.3389/fonc.2015.00248 PMC4631945

[46] Sebio A, Wilky BA, Keedy VL, Jones RL. The current landscape of early drug development for patients with sarcoma in the immunotherapy era. Future Oncol. 2018;14:1197–211. 10.2217/fon-2017-0565. 29699407 10.2217/fon-2017-0565

[47] Levental KR, Yu H, Kass L, Lakins JN, Egeblad M, Erler JT, Fong SF, Csiszar K, Giaccia A, Weninger W, et al. Matrix crosslinking forces tumor progression by enhancing integrin signaling. Cell. 2009;139:891–906. 10.1016/j.cell.2009.10.027. 19931152 10.1016/j.cell.2009.10.027 PMC2788004

[48] Lewis DM, Pruitt H, Jain N, Ciccaglione M, McCaffery JM, Xia Z, Weber K, Eisinger-Mathason TSK, Gerecht S. A Feedback loop between hypoxia and matrix stress relaxation increases oxygen-axis migration and metastasis in sarcoma. Can Res. 2019;79:1981–95. 10.1158/0008-5472.Can-18-1984. 10.1158/0008-5472.Can-18-1984 PMC6727644 30777851

[49] Han X, Wang W, He J, Jiang L, Li X. Osteopontin as a biomarker for osteosarcoma therapy and prognosis. Oncol Lett. 2019;17:2592–8. 10.3892/ol.2019.9905. 30854034 10.3892/ol.2019.9905 PMC6365895

[50] Lewis DM, Park KM, Tang V, Xu Y, Pak K, Eisinger-Mathason TS, Simon MC, Gerecht S. Intratumoral oxygen gradients mediate sarcoma cell invasion. Proc Natl Acad Sci U S A. 2016;113:9292–7. 10.1073/pnas.1605317113. 27486245 10.1073/pnas.1605317113 PMC4995943

[51] Sergi C. EPAS 1, congenital heart disease, and high altitude: disclosures by genetics, bioinformatics, and experimental embryology. 2019. Biosci Rep. 10.1042/BSR20182197. 10.1042/BSR20182197 PMC6509053 31015364

[52] Guan G, Zhang Y, Lu Y, Liu L, Shi D, Wen Y, Yang L, Ma Q, Liu T, Zhu X, et al. The HIF-1α/CXCR4 pathway supports hypoxia-induced metastasis of human osteosarcoma cells. Cancer Lett. 2015;357:254–64. 10.1016/j.canlet.2014.11.034. 25444927 10.1016/j.canlet.2014.11.034

[53] Libura J, Drukala J, Majka M, Tomescu O, Navenot JM, Kucia M, Marquez L, Peiper SC, Barr FG, Janowska-Wieczorek A, et al. CXCR4-SDF-1 signaling is active in rhabdomyosarcoma cells and regulates locomotion, chemotaxis, and adhesion. Blood. 2002;100:2597–606. 10.1182/blood-2002-01-0031. 12239174 10.1182/blood-2002-01-0031

[54] Zhang M, Zhang X. Association of MMP-2 expression and prognosis in osteosarcoma patients. Int J Clin Exp Pathol. 2015;8:14965–70. 26823829 PMC4713615

[55] Yuan X, Ma C, Li J, Li J, Yu R, Cai F, Qu G, Yu B, Liu L, Zeng D, et al. Indirect bilirubin impairs invasion of osteosarcoma cells via inhibiting the PI3K/AKT/MMP-2 signaling pathway by suppressing intracellular ROS. J Bone Oncol. 2023;39: 100472. 10.1016/j.jbo.2023.100472. 36876225 10.1016/j.jbo.2023.100472 PMC9982672

[56] Kim HS, Kim HJ, Lee MR, Han I. EMMPRIN expression is associated with metastatic progression in osteosarcoma. BMC Cancer. 2021;21:1059. 10.1186/s12885-021-08774-9. 34565336 10.1186/s12885-021-08774-9 PMC8474954

[57] Zhou J, Liu T, Wang W. Prognostic significance of matrix metalloproteinase 9 expression in osteosarcoma: a meta-analysis of 16 studies. Medicine (Baltimore). 2018;97: e13051. 10.1097/md.0000000000013051. 30383677 10.1097/md.0000000000013051 PMC6221749

[58] Laverdiere C, Hoang BH, Yang R, Sowers R, Qin J, Meyers PA, Huvos AG, Healey JH, Gorlick R. Messenger RNA expression levels of CXCR4 correlate with metastatic behavior and outcome in patients with osteosarcoma. Clin Cancer Res. 2005;11:2561–7. 10.1158/1078-0432.Ccr-04-1089. 15814634 10.1158/1078-0432.Ccr-04-1089

[59] Oda Y, Yamamoto H, Tamiya S, Matsuda S, Tanaka K, Yokoyama R, Iwamoto Y, Tsuneyoshi M. CXCR4 and VEGF expression in the primary site and the metastatic site of human osteosarcoma: analysis within a group of patients, all of whom developed lung metastasis. Mod Pathol. 2006;19:738–45. 10.1038/modpathol.3800587. 16528367 10.1038/modpathol.3800587

[60] Diomedi-Camassei F, McDowell HP, De Ioris MA, Uccini S, Altavista P, Raschellà G, Vitali R, Mannarino O, De Sio L, Cozzi DA, et al. Clinical significance of CXC chemokine receptor-4 and c-Met in childhood rhabdomyosarcoma. Clin Cancer Res. 2008;14:4119–27. 10.1158/1078-0432.Ccr-07-4446. 18593989 10.1158/1078-0432.Ccr-07-4446

[61] Yu FX, Hu WJ, He B, Zheng YH, Zhang QY, Chen L. Bone marrow mesenchymal stem cells promote osteosarcoma cell proliferation and invasion. World J Surg Oncol. 2015;13:52. 10.1186/s12957-015-0465-1. 25890096 10.1186/s12957-015-0465-1 PMC4334855

[62] Avnet S, Di Pompo G, Chano T, Errani C, Ibrahim-Hashim A, Gillies RJ, Donati DM, Baldini N. Cancer-associated mesenchymal stroma fosters the stemness of osteosarcoma cells in response to intratumoral acidosis via NF-κB activation. Int J Cancer. 2017;140:1331–45. 10.1002/ijc.30540. 27888521 10.1002/ijc.30540 PMC5272857

[63] Zaffryar-Eilot S, Hasson P. Lysyl oxidases: orchestrators of cellular behavior and ECM remodeling and homeostasis. Int J Mol Sci. 2022. 10.3390/ijms231911378. 36232685 10.3390/ijms231911378 PMC9569843

[64] Xu X, Wang B, Xu Y. Expression of lysyl oxidase in human osteosarcoma and its clinical significance: a tumor suppressive role of LOX in human osteosarcoma cells. Int J Oncol. 2013;43:1578–86. 10.3892/ijo.2013.2067. 23970168 10.3892/ijo.2013.2067

[65] Cidre-Aranaz F, Alonso J. EWS/FLI1 target genes and therapeutic opportunities in ewing sarcoma. Front Oncol. 2015;5:162. 10.3389/fonc.2015.00162. 26258070 10.3389/fonc.2015.00162 PMC4507460

[66] Huang T, Sun L, Yuan X, Qiu H. Thrombospondin-1 is a multifaceted player in tumor progression. Oncotarget. 2017;8:84546–58. 10.18632/oncotarget.19165. 29137447 10.18632/oncotarget.19165 PMC5663619

[67] Robinet A, Emonard H, Banyai L, Laronze JY, Patthy L, Hornebeck W, Bellon G. Collagen-binding domains of gelatinase A and thrombospondin-derived peptides impede endocytic clearance of active gelatinase A and promote HT1080 fibrosarcoma cell invasion. Life Sci. 2008;82:376–82. 10.1016/j.lfs.2007.11.018. 18222489 10.1016/j.lfs.2007.11.018

[68] Decker S, van Valen F, Vischer P. Adhesion of osteosarcoma cells to the 70-kDa core region of thrombospondin-1 is mediated by the alpha 4 beta 1 integrin. Biochem Biophys Res Commun. 2002;293:86–92. 10.1016/s0006-291x(02)00180-8. 12054567 10.1016/s0006-291x(02)00180-8

[69] Dossi R, Frapolli R, Di Giandomenico S, Paracchini L, Bozzi F, Brich S, Castiglioni V, Borsotti P, Belotti D, Uboldi S, et al. Antiangiogenic activity of trabectedin in myxoid liposarcoma: involvement of host TIMP-1 and TIMP-2 and tumor thrombospondin-1. Int J Cancer. 2015;136:721–9. 10.1002/ijc.29023. 24917554 10.1002/ijc.29023

[70] Ng F, Boucher S, Koh S, Sastry KS, Chase L, Lakshmipathy U, Choong C, Yang Z, Vemuri MC, Rao MS, et al. PDGF, TGF-beta, and FGF signaling is important for differentiation and growth of mesenchymal stem cells (MSCs): transcriptional profiling can identify markers and signaling pathways important in differentiation of MSCs into adipogenic, chondrogenic, and osteogenic lineages. Blood. 2008;112:295–307. 10.1182/blood-2007-07-103697. 18332228 10.1182/blood-2007-07-103697

[71] Yang G, Yuan J, Li K. EMT transcription factors: implication in osteosarcoma. Med Oncol. 2013;30:697. 10.1007/s12032-013-0697-2. 23975634 10.1007/s12032-013-0697-2

[72] Lamora A, Talbot J, Bougras G, Amiaud J, Leduc M, Chesneau J, Taurelle J, Stresing V, Le Deley MC, Heymann MF, et al. Overexpression of smad7 blocks primary tumor growth and lung metastasis development in osteosarcoma. Clin Cancer Res. 2014;20:5097–112. 10.1158/1078-0432.Ccr-13-3191. 25107916 10.1158/1078-0432.Ccr-13-3191

[73] Babar Q, Saeed A, Murugappan S, Dhumal D, Tabish T, Thorat ND. Promise of dostarlimab in cancer therapy: advancements and cross-talk considerations. Drug Discov Today. 2023;28: 103577. 10.1016/j.drudis.2023.103577. 37004983 10.1016/j.drudis.2023.103577

[74] Huang Y, Yang Y, Gao R, Yang X, Yan X, Wang C, Jiang S, Yu L. RLIM interacts with Smurf2 and promotes TGF-beta induced U2OS cell migration. Biochem Biophys Res Commun. 2011;414:181–5. 10.1016/j.bbrc.2011.09.053. 21945933 10.1016/j.bbrc.2011.09.053

[75] Kunita A, Kashima TG, Ohazama A, Grigoriadis AE, Fukayama M. Podoplanin is regulated by AP-1 and promotes platelet aggregation and cell migration in osteosarcoma. Am J Pathol. 2011;179:1041–9. 10.1016/j.ajpath.2011.04.027. 21801875 10.1016/j.ajpath.2011.04.027 PMC3157255

[76] Lamora A, Mullard M, Amiaud J, Brion R, Heymann D, Redini F, Verrecchia F. Anticancer activity of halofuginone in a preclinical model of osteosarcoma: inhibition of tumor growth and lung metastases. Oncotarget. 2015;6:14413–27. 10.18632/oncotarget.3891. 26015407 10.18632/oncotarget.3891 PMC4546476

[77] Hahm KB, Cho K, Lee C, Im YH, Chang J, Choi SG, Sorensen PH, Thiele CJ, Kim SJ. Repression of the gene encoding the TGF-beta type II receptor is a major target of the EWS-FLI1 oncoprotein. Nat Genet. 1999;23:222–7. 10.1038/13854. 10508522 10.1038/13854

[78] Pardali E, van der Schaft DW, Wiercinska E, Gorter A, Hogendoorn PC, Griffioen AW, ten Dijke P. Critical role of endoglin in tumor cell plasticity of Ewing sarcoma and melanoma. Oncogene. 2011;30:334–45. 10.1038/onc.2010.418. 20856203 10.1038/onc.2010.418

[79] Katz LH, Li Y, Chen JS, Munoz NM, Majumdar A, Chen J, Mishra L. Targeting TGF-beta signaling in cancer. Expert Opin Ther Targets. 2013;17:743–60. 10.1517/14728222.2013.782287. 23651053 10.1517/14728222.2013.782287 PMC3745214

[80] Yang J, Yang D, Sun Y, Sun B, Wang G, Trent JC, Araujo DM, Chen K, Zhang W. Genetic amplification of the vascular endothelial growth factor (VEGF) pathway genes, including VEGFA, in human osteosarcoma. Cancer. 2011;117:4925–38. 10.1002/cncr.26116. 21495021 10.1002/cncr.26116 PMC3465081

[81] Tsai HC, Su HL, Huang CY, Fong YC, Hsu CJ, Tang CH. CTGF increases matrix metalloproteinases expression and subsequently promotes tumor metastasis in human osteosarcoma through down-regulating miR-519d. Oncotarget. 2014;5:3800–12. 10.18632/oncotarget.1998. 25003330 10.18632/oncotarget.1998 PMC4116521

[82] Tsai HC, Su HL, Huang CY, Fong YC, Hsu CJ, Tang CH. Correction: CTGF increases matrix metalloproteinases expression and subsequently promotes tumor metastasis in human osteosarcoma through down-regulating miR-519d. Oncotarget. 2020;11:492. 10.18632/oncotarget.27459. 32064054 10.18632/oncotarget.27459 PMC6996914

[83] Wang LH, Tsai HC, Cheng YC, Lin CY, Huang YL, Tsai CH, Xu GH, Wang SW, Fong YC, Tang CH. CTGF promotes osteosarcoma angiogenesis by regulating miR-543/angiopoietin 2 signaling. Cancer Lett. 2017;391:28–37. 10.1016/j.canlet.2017.01.013. 28108312 10.1016/j.canlet.2017.01.013

[84] Hou CH, Yang RS, Tsao YT. Connective tissue growth factor stimulates osteosarcoma cell migration and induces osteosarcoma metastasis by upregulating VCAM-1 expression. Biochem Pharmacol. 2018;155:71–81. 10.1016/j.bcp.2018.06.015. 29909077 10.1016/j.bcp.2018.06.015

[85] Tsai HC, Huang CY, Su HL, Tang CH. CTGF increases drug resistance to paclitaxel by upregulating survivin expression in human osteosarcoma cells. Biochim Biophys Acta. 2014;1843:846–54. 10.1016/j.bbamcr.2014.01.007. 24462773 10.1016/j.bbamcr.2014.01.007

[86] Liu SC, Chuang SM, Hsu CJ, Tsai CH, Wang SW, Tang CH. CTGF increases vascular endothelial growth factor-dependent angiogenesis in human synovial fibroblasts by increasing miR-210 expression. Cell Death Dis. 2014;5: e1485. 10.1038/cddis.2014.453. 25341039 10.1038/cddis.2014.453 PMC4649533

[87] Huang JJ, Blobe GC. Dichotomous roles of TGF-β in human cancer. Biochem Soc Trans. 2016;44:1441–54. 10.1042/bst20160065. 27911726 10.1042/bst20160065 PMC5682628

[88] Pickup M, Novitskiy S, Moses HL. The roles of TGFβ in the tumour microenvironment. Nat Rev Cancer. 2013;13:788–99. 10.1038/nrc3603. 24132110 10.1038/nrc3603 PMC4025940

[89] Heymann MF, Lezot F, Heymann D. The contribution of immune infiltrates and the local microenvironment in the pathogenesis of osteosarcoma. Cell Immunol. 2019;343: 103711. 10.1016/j.cellimm.2017.10.011. 29117898 10.1016/j.cellimm.2017.10.011

[90] Kawano M, Itonaga I, Iwasaki T, Tsuchiya H, Tsumura H. Anti-TGF-β antibody combined with dendritic cells produce antitumor effects in osteosarcoma. Clin Orthop Relat Res. 2012;470:2288–94. 10.1007/s11999-012-2299-2. 22415727 10.1007/s11999-012-2299-2 PMC3392369

[91] Gao Y, Souza-Fonseca-Guimaraes F, Bald T, Ng SS, Young A, Ngiow SF, Rautela J, Straube J, Waddell N, Blake SJ, et al. Tumor immunoevasion by the conversion of effector NK cells into type 1 innate lymphoid cells. Nat Immunol. 2017;18:1004–15. 10.1038/ni.3800. 28759001 10.1038/ni.3800

[92] Chang KK, Yoon C, Yi BC, Tap WD, Simon MC, Yoon SS. Platelet-derived growth factor receptor-α and -β promote cancer stem cell phenotypes in sarcomas. Oncogenesis. 2018;7:47. 10.1038/s41389-018-0059-1. 29915281 10.1038/s41389-018-0059-1 PMC6006341

[93] Ehnman M, Missiaglia E, Folestad E, Selfe J, Strell C, Thway K, Brodin B, Pietras K, Shipley J, Östman A, et al. Distinct effects of ligand-induced PDGFRα and PDGFRβ signaling in the human rhabdomyosarcoma tumor cell and stroma cell compartments. Can Res. 2013;73:2139–49. 10.1158/0008-5472.Can-12-1646. 10.1158/0008-5472.Can-12-1646 PMC3672973 23338608

[94] Benini S, Baldini N, Manara MC, Chano T, Serra M, Rizzi S, Lollini PL, Picci P, Scotlandi K. Redundancy of autocrine loops in human osteosarcoma cells. Int J Cancer. 1999;80:581–8. 10.1002/(sici)1097-0215(19990209)80:4%3c581::aid-ijc16%3e3.0.co;2-o. 9935160 10.1002/(sici)1097-0215(19990209)80:4<581::aid-ijc16>3.0.co;2-o

[95] Pietras K, Ostman A, Sjöquist M, Buchdunger E, Reed RK, Heldin CH, Rubin K. Inhibition of platelet-derived growth factor receptors reduces interstitial hypertension and increases transcapillary transport in tumors. Can Res. 2001;61:2929–34. 11306470

[96] Ehnman M, Östman A. Therapeutic targeting of platelet-derived growth factor receptors in solid tumors. Expert Opin Investig Drugs. 2014;23:211–26. 10.1517/13543784.2014.847086. 24206431 10.1517/13543784.2014.847086

[97] Taniguchi E, Nishijo K, McCleish AT, Michalek JE, Grayson MH, Infante AJ, Abboud HE, Legallo RD, Qualman SJ, Rubin BP, et al. PDGFR-A is a therapeutic target in alveolar rhabdomyosarcoma. Oncogene. 2008;27:6550–60. 10.1038/onc.2008.255. 18679424 10.1038/onc.2008.255 PMC2813858

[98] Horst D, Ustanina S, Sergi C, Mikuz G, Juergens H, Braun T, Vorobyov E. Comparative expression analysis of Pax3 and Pax7 during mouse myogenesis. Int J Dev Biol. 2006;50:47–54. 10.1387/ijdb.052111dh. 16323077 10.1387/ijdb.052111dh

[99] Zwerner JP, May WA. Dominant negative PDGF-C inhibits growth of Ewing family tumor cell lines. Oncogene. 2002;21:3847–54. 10.1038/sj.onc.1205486. 12032822 10.1038/sj.onc.1205486

[100] Bennani-Baiti IM, Cooper A, Lawlor ER, Kauer M, Ban J, Aryee DN, Kovar H. Intercohort gene expression co-analysis reveals chemokine receptors as prognostic indicators in Ewing’s sarcoma. Clin Cancer Res. 2010;16:3769–78. 10.1158/1078-0432.Ccr-10-0558. 20525755 10.1158/1078-0432.Ccr-10-0558 PMC2905506

[101] Berning P, Schaefer C, Clemens D, Korsching E, Dirksen U, Potratz J. The CXCR4 antagonist plerixafor (AMD3100) promotes proliferation of Ewing sarcoma cell lines in vitro and activates receptor tyrosine kinase signaling. Cell Commun Signal. 2018;16:21. 10.1186/s12964-018-0233-2. 29776413 10.1186/s12964-018-0233-2 PMC5960216

[102] Huang F, Hurlburt W, Greer A, Reeves KA, Hillerman S, Chang H, Fargnoli J, Graf Finckenstein F, Gottardis MM, Carboni JM. Differential mechanisms of acquired resistance to insulin-like growth factor-i receptor antibody therapy or to a small-molecule inhibitor, BMS-754807, in a human rhabdomyosarcoma model. Can Res. 2010;70:7221–31. 10.1158/0008-5472.Can-10-0391. 10.1158/0008-5472.Can-10-0391 20807811

[103] Roberts BK, Li DI, Somerville C, Matta B, Jha V, Steinke A, Brune Z, Blanc L, Soffer SZ, Barnes BJ. IRF5 suppresses metastasis through the regulation of tumor-derived extracellular vesicles and pre-metastatic niche formation. Sci Rep. 2024;14:15557. 10.1038/s41598-024-66168-w. 38969706 10.1038/s41598-024-66168-w PMC11226449

[104] Chen Q, Xing C, Zhang Q, Du Z, Kong J, Qian Z. PDE1B, a potential biomarker associated with tumor microenvironment and clinical prognostic significance in osteosarcoma. Sci Rep. 2024;14:13790. 10.1038/s41598-024-64627-y. 38877061 10.1038/s41598-024-64627-y PMC11178771

[105] Al-Shehri A, Bakhashab S. Oncogenic long noncoding RNAs in prostate cancer, osteosarcoma, and metastasis. Biomedicines. 2023. 10.3390/biomedicines11020633. 36831169 10.3390/biomedicines11020633 PMC9953056

[106] Fan H, Zhou Y, Zhang Z, Zhou G, Yuan C. ROR1-AS1: a meaningful long noncoding RNA in oncogenesis. Mini Rev Med Chem. 2024. 10.2174/0113895575294482240530154620. 38859780 10.2174/0113895575294482240530154620

[107] Wu X, Yan L, Liu Y, Shang L. LncRNA ROR1-AS1 accelerates osteosarcoma invasion and proliferation through modulating miR-504. Aging (Albany NY). 2020;13:219–27. 10.18632/aging.103498. 33401251 10.18632/aging.103498 PMC7835057

[108] Perkins RS, Murray G, Suthon S, Davis L, Perkins NB 3rd, Fletcher L, Bozzi A, Schreiber SL, Lin J, Laxton S, et al. WNT5B drives osteosarcoma stemness, chemoresistance and metastasis. Clin Transl Med. 2024;14: e1670. 10.1002/ctm2.1670. 38689429 10.1002/ctm2.1670 PMC11061378

[109] Chu Y, Nayyar G, Jiang S, Rosenblum JM, Soon-Shiong P, Safrit JT, Lee DA, Cairo MS. Combinatorial immunotherapy of N-803 (IL-15 superagonist) and dinutuximab with ex vivo expanded natural killer cells significantly enhances in vitro cytotoxicity against GD2(+) pediatric solid tumors and in vivo survival of xenografted immunodeficient NSG mice. J Immunother Cancer. 2021. 10.1136/jitc-2020-002267. 34244307 10.1136/jitc-2020-002267 PMC8268924

[110] Zhu T, Han J, Yang L, Cai Z, Sun W, Hua Y, Xu J. Immune microenvironment in osteosarcoma: components, therapeutic strategies and clinical applications. Front Immunol. 2022;13: 907550. 10.3389/fimmu.2022.907550. 35720360 10.3389/fimmu.2022.907550 PMC9198725

